# The Immune Response of the Invasive Golden Apple Snail to a Nematode-Based Molluscicide Involves Different Organs

**DOI:** 10.3390/biology9110371

**Published:** 2020-10-30

**Authors:** Alice Montanari, Giulia Bergamini, Agnese Ferrari, Anita Ferri, Milena Nasi, Roberto Simonini, Davide Malagoli

**Affiliations:** 1 The Institute of Interdisciplinary Research in Human and Molecular Biology (IRIBHM), Free University of Brussels (ULB), 1070 Brussels, Belgium; Alice.Montanari@ulb.be; 2 Department of Life Sciences, University of Modena and Reggio Emilia, 41125 Modena, Italy; giulia.bergamini@unimore.it (G.B.); agnese.ferrari@unimore.it (A.F.); 224420@studenti.unimore.it (A.F.); roberto.simonini@unimore.it (R.S.); 3 Department of Surgery, Medicine, Dentistry and Morphological Sciences, University of Modena and Reggio Emilia, 41125 Modena, Italy; milena.nasi@unimore.it

**Keywords:** eco-immunology, hemocytes, immunity, invertebrate, mollusk, molluscicide, pest control, *Phasmarhabditis hermaphrodita*, *Pomacea canaliculata*

## Abstract

**Simple Summary:**

Sustainable solutions to the spreading of invasive species are difficult to find due to the absence of biological information about basic immune mechanisms of the target pests. Here, we present evidence of the effects of a commercially available roundworm, *Phasmarhabditis hermaphrodita*, against the invasive apple snail *Pomacea canaliculata*. The effects are principally evaluated in terms of snail survival and immune activation. Via molecular and microscopy-based approaches, we demonstrate that dosage and temperature are critical in determining the effects of the roundworm, and that the apple snail response to this immune challenge involves different organs. To our knowledge, these findings are the first demonstration that a *P. hermaphrodita*-based molluscicide can effectively kill *P. canaliculata* and that the snail can mount a multi-organ response against this pathogenic roundworm.

**Abstract:**

The spreading of alien and invasive species poses new challenges for the ecosystem services, the sustainable production of food, and human well-being. Unveiling and targeting the immune system of invasive species can prove helpful for basic and applied research. Here, we present evidence that a nematode (*Phasmarhabditis hermaphrodita*)-based molluscicide exerts dose-dependent lethal effects on the golden apple snail, *Pomacea canaliculata*. When used at 1.7 g/L, this biopesticide kills about 30% of snails within one week and promotes a change in the expression of *Pc-bpi*, an orthologue of mammalian bactericidal/permeability increasing protein (BPI). Changes in *Pc-bpi* expression, as monitored by quantitative PCR (qPCR), occurred in two immune-related organs, namely the anterior kidney and the gills, after exposure at 18 and 25 °C, respectively. Histological analyses revealed the presence of the nematode in the snail anterior kidney and the gills at both 18 and 25 °C. The mantle and the central nervous system had a stable *Pc-bpi* expression and seemed not affected by the nematodes. Fluorescence in situ hybridization (FISH) experiments demonstrated the expression of *Pc-bpi* in circulating hemocytes, nurturing the possibility that increased *Pc-bpi* expression in the anterior kidney and gills may be due to the hemocytes patrolling the organs. While suggesting that *P. hermaphrodita*-based biopesticides enable the sustainable control of *P. canaliculata* spread, our experiments also unveiled an organ-specific and temperature-dependent response in the snails exposed to the nematodes. Overall, our data indicate that, after exposure to a pathogen, the snail *P. canaliculata* can mount a complex, multi-organ innate immune response.

## 1. Introduction

Indexed among the worst invasive species in the world, the freshwater snail *Pomacea canaliculata* has gained significant attention within the scientific community [1,2]. This is principally due to the different typologies of damages that the snail can cause to human economy and health. With regard to human health, *P. canaliculata* is the intermediate vector of a dangerous nematode, *Angiostrongylus cantonensis*, which causes eosinophilic encephalitis in humans and other mammals, such as dogs and horses. Although several restrictions have been enforced over the circulation of *P. canaliculata*, in the last few years the spreading of this pest has been widening worldwide [3]. With the idea of targeting the golden apple snail immune system as a tool to control its distribution, we sought to investigate the basic immune components in this snail. As all lophotrochozoa, *P. canaliculata* possesses an exclusively innate immune system [4], which, together with specific anatomical and physiological features [1], contributes to the snail’s notorious adaptability. Morphological, ultrastructural, and flow cytometry studies converged towards the conclusion that the snail immune cellular component is principally represented by circulating hemocytes, divided into Group I and Group II hemocytes on the basis of their size. Group II hemocytes can be further divided into agranular and granular hemocytes [5,6]. Beside circulating hemocytes, other resident hemocytes or hemocyte-like cells have been described in the ampulla, a saccular organ positioned along the main circulatory flux [7,8] and in the posterior kidney [9]. In *P. canaliculata* and other gastropods, the presence of granular hemocytes and/or immune-related functions have also been identified in the anterior kidney [8], the gills [10], the mantle [8,11], and the pedal ganglia [12]. Like for other freshwater snails, *P. canaliculata* hematopoiesis takes place in the pericardial cavity, which contains replicating cells with a morphology similar to that of circulating hemocytes [7]. Recently, 5-bromo-2′-deoxyuridine accumulation has also been detected in the hemocyte islets of the posterior kidney after yeast injection [9], thus highlighting another potential district for hemocyte replication. While Group II and renal hemocytes have been demonstrated to perform phagocytosis, nodulation in response to worm-like parasites has also been documented. The mammalian parasite *A. cantonensis* finds the golden apple snail a suitable intermediate host. This parasite, whose diffusion is a cause for concern due to its wide distribution [13], triggers the formation of recognizable nodules in the infected snails [14], although the involvement of hemocytes in this process has not been documented. While our knowledge of the humoral components of the *P. canaliculata* immune system is increasing thanks to genomics and proteomics studies [1,15,16], the analysis of their potential role as mediators of the immune response against pathogens, especially nematodes, is still in its infancy [8].

On these premises, we sought to test the effects of Nemaslug^®^, a commercial product based on the nematode *Phasmarhabditis hermaphrodita*, a worm lethal for terrestrial snails and slugs [17,18] but harmless to other aquatic snails [19]. The aims of our study were to assess the susceptibility of *P. canaliculata* to this biological control agent and to identify and analyze the expression of bactericidal/permeability-increasing protein (BPI), a conserved immune-related soluble mediator, after the exposure to the parasitic nematode. BPI is a member of a structurally related family of lipid-binding proteins that includes lipopolysaccharide (LPS)-binding protein (LBP), cholesteryl ester transfer proteins (CETPs) and phospholipid transfer proteins (PLTPs) [20,21,22]. In humans, BPI is stored in the granules of neutrophils and in mucosal epithelial cells [23], it displays opsonizing activity, and its secretion neutralizes LPS inflammatory properties [24]. The presence of *bpi* genes and transcripts has been reported in mollusks [25], but, so far, no information has been available for *P. canaliculata*.

Overall, by studying the effects of a nematode-based molluscicide and the related changes in *bpi* expression and by matching our results with morphological analyses, we provide novel information for the sustainable control of *P. canaliculata* diffusion and for a better understanding of the immune functions in this molluscan model.

## 2. Materials and Methods

### 2.1. Animals

*P. canaliculata* specimens were reared and bred in tap water at 25 ± 1 °C, dark/light cycle of 12 h. Two-thirds of the water contained in each tank was replaced twice a week and, immediately after water change, animals were fed with mixed types of green salad, suitable for human consumption. Adults with a shell diameter between 35 and 50 mm were used in the experimental protocols described below. To avoid contamination with undigested food or digestion products, animals were starved for 48 h before hemolymph withdrawal [7].

### 2.2. Nemaslug^®^ Exposure

To evaluate the effects of the nematode *P. hermaphrodita* on *P. canaliculata* viability and food consumption, animals were incubated in 5 L of water with three different concentrations of Nemaslug^®^ (BASF SE, Ludwigshafen, Germany). Each concentration was tested at three different temperatures. In more detail, 15 animals were exposed to 0.17 or 1.7 or 17 g/L (approximately corresponding to 25,500, 255,000, and 2,550,000 worms/L, respectively), at the three different temperatures of 18, 25, and 30 °C. The intermediate concentration we applied (i.e., 1.7 g/L) corresponds to the dose recommended by the supplier for distribution in the open field. The temperature extremes were chosen on the basis of observations concerning the limit temperature for growth and reproductive activity of *P. canaliculata* [26], and 25 °C is the optimal T at which this snail is usually bred in the laboratory [5,26]. The experimental temperatures were also selected using as reference the published parameters for the optimal breeding and survival of the nematode *P. hermaphrodita,* which indicate 20 °C as optimal temperature and 25 °C as the upper limit for the survival of hermaphrodite females [27]. Treatments lasted one week, with water and Nemaslug^®^ changed daily. The vitality of the nematodes was checked by inspecting the tank waters under a dissection microscope after each water change. The evaluations of the survival and feeding of snails on the 10 g of salad were performed twice a day during the experiments.

For qPCR and histological analyses, the animals (*N* = 6 for qPCR; *N* = 3 for histology) were exposed to the intermediate concentration of Nemaslug^®^ (i.e., 1.7 g/L) for each one of the three different temperatures. Snails were exposed to the nematode-based molluscicide for 24 h in order to detect changes in gene expression during the first phase of the immune response against the nematode.

Control snails were kept in the same conditions of the treated specimens (tank size and temperature) but in the absence of Nemaslug^®^. The same number of control and treated animals were used for qPCR and histological analyses. Given the equal sample sizes and the lack of repeated values, the number of replicates was proportioned and adequate for the adopted statistical tests (see below).

### 2.3. Animal Dissection and Organ Collection for RNA Extraction and Histological Analysis

The samples for the histological analysis were collected from snails exposed to 1.7 g/L Nemaslug^®^ at the end of the experimental week. After a 20 min anesthesia in ice, the snails were quickly dissected under a dissection microscope to identify the presence of living nematodes inside the body cavity and selected organs (namely anterior kidney, gills, and mantle, and the entire cephalic and podalic ganglia). For each organ, 20 mg of tissue were processed for RNA extraction (see the section on qPCR experiments) or immediately fixed for histological analysis.

### 2.4. Pomacea canaliculata BPI (Pc-BPI) Identification, Sequence Analysis, and Primer Identification

In the absence of established readouts for *P. canaliculata* immune system activation, by interrogating an organ-specific RNAseq database from Sánchez-Alvarado Laboratory at the Stowers Institute for Medical Research (Kansas City, MO, USA) [2], we identified the presence and expression of a putative *bpi* gene in our target organs. The sequence of the candidate gene was further confirmed by BLAST analysis against the available *P. canaliculata* genome [1,2] and through multiple alignment with molluscan *lbp/bpi* genes available through the National Center for Biotechnology Information (NCBI) nucleotide and protein databases. The presence of the LBP/BPI-characteristic C-terminal and N-terminal domains in the putative peptide was ascertained using HMMER software https://www.ebi.ac.uk/Tools/hmmer/, HmmerWeb version 2.41.1) and then confirmed by analysis of domain similarity in the Pfam protein collection (https://pfam.xfam.org/, version 33.1).

As a further confirmation step, we also compared the *P. canaliculata* BPI (*Pc*-BPI) putative protein sequence with invertebrate BPI/LBPs and vertebrate BPIs, with the multiple alignment tool, MUSCLE, with default setting parameters (http://www.ebi.ac.uk/Tools/msa/muscle/, version 3.6). Results were visualized using the “Multiple align show” tool in The Sequence Manipulation Suite (http://www.bioinformatics.org/sms/version 1) that highlights identical (yellow) and similar (green) amino acids.

### 2.5. qPCR Protocol

After dissection and weighing, about 20 mg of tissue were either stored at −80 °C or immediately processed for RNA extraction. Total RNA purification was performed on dissected organs using a Quick-RNA™ Miniprep Kit (Zymo Research, Freiburg, Germany) following the manufacturer’s protocol. All RNA samples were then checked for purity and quantified using Qubit^®^ RNA HS Assays (Invitrogen, Carlsbad, CA, USA) and a NanoDrop™ ND1000 Spectrophotometer (Thermo Fisher Scientific, Waltham, MA, USA).

The mRNA was reverse transcribed to cDNA using an iScript^®^ cDNA Synthesis Kit (Bio-Rad Laboratories, Inc., Hercules, CA, USA) according to the manufacturer’s instructions.

The qPCR reaction was performed using a SsoAdvanced™ Universal SYBR^®^ Green Supermix (Bio-Rad Laboratories, Inc.) with primers amplifying a fragment of the housekeeping gene ribosomal protein L5 (rpL5) and the target gene bpi, i.e., rpL5_F 5′ -CGTATGCCAGAATTGAGGGT-3′, rpL5_R 5′ -CAACATCCAAGTATGCACGG- 3′, bpi_F 5′ -GCTATTGGGCCTTTGAATGA- 3′, and bpi_R 5′ -GGAGGGGGAAACCTCTACTG- 3′, following instructions provided by the manufacturer. The applied thermal profile was as follows: 95 °C for 10 min (1 cycle), 95 °C for 2 min, and 60 °C for 30 s (30 cycles). All the reactions were performed in triplicate on a CFX96 Touch Real-time PCR detection system (Bio-Rad Laboratories, Inc.). Once the amplification reaction was completed, the melting curves were inspected for all the amplicons.

### 2.6. Slide Preparation and Staining for Histological Analysis

After dissection, the organs were fixed in ice cold 4% buffered paraformaldehyde (pFA) in 0.5× freshwater snail solution (FSS) [5]. After overnight (ON) fixation at 4 °C, the samples were dehydrated with a standard alcoholic gradient and embedded in EM-400 embedding medium (Leica, Wetzlar, Germany) in a PATHOS Delta hybrid tissue processor (Milestone Medical, Sorisole, Italy). The embedded specimens were then cut into 7 µm slices using a rotary microtome and mounted on SureBond™ Charged Microscope Slides (Avantik, Springfield, NJ, USA).

For histological analysis of control and nematode-exposed snails, the slides were stained accordingly the following Masson’s trichrome protocol. After 45 min post-fixation in Bouin’s solution at 60 °C for enhancing the differential staining of the tissue components, the slides were washed for 3 min under warm running tap water, rinsed in deionized water, stained with Weigert’s hematoxylin for 10 min, washed under running tap water for 5 min, and then rinsed in deionized water. Slides were then stained with Biebrich scarlet-acid fuchsin for 2 min, rinsed with deionized water, placed in phosphomolybdic/phosphotungstic acid solution for 10 min, and finally placed in aniline blue for 5 min. Slide staining was completed with 1% acetic acid for 1 min and dehydrated with standard alcohol scale before being mounted in organic mounting medium.

### 2.7. Hemolymph Withdrawal and Cytocentrifugation

Hemolymph was extracted from control snails by applying gentle and continuous pressure onto the operculum and collecting the released hemolymph in a 10 mL tube, kept in ice [5]. About 2 mL of hemolymph were collected from each animal. For each slide, 150 μL of freshly collected hemolymph were cytocentrifuged at 400 rpm for 3 min onto slides using Cytospin II™ (Shandon Inc., Pittsburgh, PA, USA). The hemocytes were immediately fixed with 4% buffered pFA in 1× PBS for 3 min at room temperature (RT). The fixation was blocked with a 5 min wash in 1× PBS, before starting the in situ hybridization protocol.

### 2.8. Riboprobe Synthesis

*Pc-bpi* primers for the synthesis of a 500–700 base pair (bp) riboprobe were designed with Primer3Plus (http://www.bioinformatics.nl/cgi-bin/primer3plus/primer3plus.cgi version 3.1.1). At each 5′ end of the primers, a sequence for the oriented cloning into the T4p plasmid was added. Amplifications were performed using Phusion High Fidelity DNA Polymerase (M0530S, NEB, Ipswich, MA, USA) and PCR conditions were set as follows: 94 °C for 15 s, 56 °C for 30 s, and 72 °C for 1 min for 35 cycles.

The PCR products were purified from agarose gel with a QIAquick Gel Extraction Kit (QIAGEN N.V., Hilden, Germany) according to the manufacturer’s protocol, and their concentration was checked with a NanoDrop™ ND1000 Spectrophotometer (Thermo Fisher Scientific).

For the ligation step, we used a Gibson Assembly^®^ Cloning kit (E5510S, New England Biolabs Inc, Ipswich, MA, USA) according to the manufacturer’s protocol. Transformation of competent bacteria (*Escherichia coli*, DH5α strain) was performed by heat shock, before incubation in Luria–Bertani (LB) broth at 37 °C for 1 h shaking and plating onto LB supplemented with 50 mg/mL Kanamycin agar plates.

After incubation ON at 37 °C, colonies were pricked and screened by PCR reactions for containing the 500–700 bp gene-specific amplified insert. Positive colonies were further grown in liquid cultures (LB broth + 50 mg/mL Kanamycin ON at 37 °C). These liquid cultures underwent plasmid extraction and purification using a QIAprep^®^ Miniprep Kit (QIAGEN, Hilden, Germany) according to the standard manufacturer’s instructions. For each developed probe, one plasmid was submitted for sequencing. A further PCR reaction with the T4p primer was then performed (94 °C for 15 s, 56 °C for 30 s and 72 °C for 40 s, 35 cycles) to collect the template for the synthesis of digoxigenin (DIG)-labelled riboprobes. In vitro transcription protocol was performed with 1× Transcript. Opt. Buffer (Promega, Madison, WI, USA), 1× DIG-NTPs RNA Labelling Mix (Roche, Basel, Switzerland), 2.4 U/µl RNase inhibitor (Promega), and 1.6 U/µl T7 RNA polymerase (Promega). Between 0.8 and 1 µg of purified PCR product were used as a template for each reaction. Labelled riboprobes were purified by a standard precipitation in ice-cold 100% ethanol and ammonium acetate, washed in 75% ethanol, and diluted in deionized formamide.

### 2.9. FISH Protocol

FISH experiments were performed on freshly cytocentrifuged hemocyte spots from 3 control animals, following a modified King and Newmark’s [28] protocol. Fixed hemocytes were clarified in bleaching solution (1.2% H_2_O_2_ and 5% formamide in 0.5× saline sodium citrate (SSC)) for 1 h. After bleaching, the slides were rinsed twice with 1× PBS-Tween^®^ 20 (Bio-Rad Laboratories, Inc.) 0.1% (PBST), permeabilized with 3 µg/mL proteinase K in PBST for 10 min, and post-fixed in 4% PFA for 10 min.

Post-fixation, the slides were incubated first in 1:1 1× PBS:Pre-Hybridization Solution (HS) (50% formamide, 1% Tween^®^ 20, 5× SSC, 1× Denhardt’s solution, 50 mM dithiothreitol, 1 mg/mL torula yeast RNA, and 100 µg/mL heparin) for 10 min at RT and then in pre-HS for 1 h at 58 °C. Slides were then incubated ON at 58 °C with *Pc*-BPI riboprobes (50 ng/µl) diluted 1:500 in HS. Negative control slides were prepared as indicated above but in the absence of riboprobes.

After hybridization, the slides were washed 2 times with Wash-HS (50% formamide, 1% Tween^®^ 20, 5× SSC, and 1× Denhardt’s solution) for 10 min at 58 °C, then 2 times with 1:1 Wash-HS:2×SSC for 10 min at 58 °C, 3 times with 2×SSC + 0.1% Tween^®^ 20 for 10 min at 58 °C, three times with 0.2×SSC + 0.1% Tween^®^ 20 for 10 min at 58 °C, and finally two times with maleic buffer/Tween 20 (MABT) (11.61 g/L maleic acid, 150 mM NaCl, 0.1% Tween 20) at RT for 10 min. Samples were then incubated in blocking solution (BS) (5% horse serum and 0.5% Roche Western Blocking Reagent in MABT) for 1 h at RT, and with 1:1000 anti-DIG-peroxidase-conjugated (POD) Ab (16620900, Roche) in filtered BS, ON at 4 °C. Following the ON incubation, we performed 6 subsequent washes of 20 min each in MABT at RT. After washes, slides were incubated with Tyramide Development Solution (TDS), consisting of 1:2000 fluorescein amidite (FAM)-tyramide in borate buffer (2 M NaCl, 0.1 M boric acid) plus 0.06‰ of hydrogen peroxide for 1 h, at RT. After incubation, the remaining TDS was removed with 2 washes in PBST at RT. Nuclei were counterstained with DAPI directly added to Scale A2 aqueous and clarifying mounting medium (4 M urea, 0.1% Triton X-100, 20% glycerol, and 2.5% 1,4-diazabicyclo [2.2.2]octane (DABCO)).

### 2.10. Histology and FISH Imaging

Following Masson’s trichrome staining, images were acquired using a BH2 microscope (Olympus, Tokyo, Japan) equipped with a DS-5M-L1 digital camera system (Nikon, Tokyo, Japan)

Images of FISH from hemocyte spots were taken with a Leica TCS SP8 confocal microscope (Leica Microsystems Srl, Milan, Italy) at the Centro Interdipartimentale Grandi Strumenti (C.I.G.S.) of the University of Modena and Reggio Emilia, Modena, Italy. Positive cell detection was performed from random microscopic fields using the ScanR 3.1 software (Olympus Life Science, Tokyo, Japan) and selecting approximately 650 hemocytes.

All other image processing was performed with the ImageJ software (version number 1.52p, NIH, Bethesda, MD, USA) [29].

### 2.11. Statistical Analysis

Statistical significance of observed mortality after one-week treatment was assessed using Fisher’s exact test, and the expected mortality, in the case of a lack of nematode effects, was set at 0%. Relative quantification of qPCR data was obtained through the 2^-(ΔΔCt)^ method [30]. The changes in mRNA levels between control and treated animals were analyzed with the non-parametric Mann–Whitney U test. *p*-values ≤ 0.05 were considered as statistically significant.

## 3. Results

### 3.1. Effect of Nemaslug^®^ on P. canaliculata Survival and Feeding

**Figure 1:**
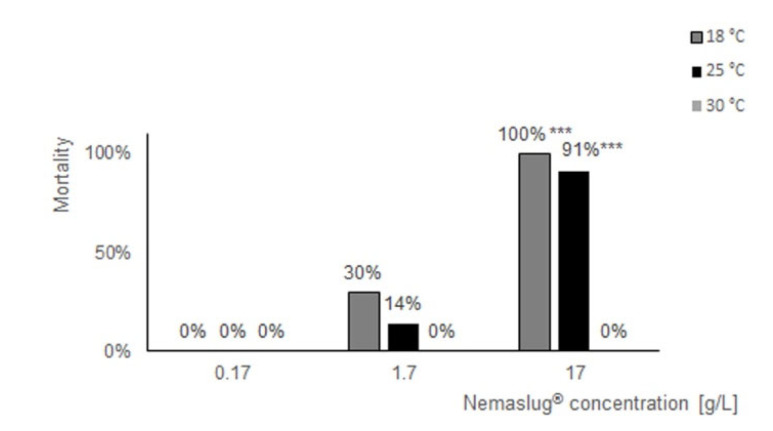
Concentration and temperature-dependent effects of the Nemaslug^®^ molluscicide. The bar chart reports the mortality of apple snail exposed to the *Phasmarhabditis hermaphrodita* worm, that increases with Nemaslug^®^ concentration. At 18 (dark gray) and 25 (black) °C, lethal effects on the apple snails were observed, whereas at 30 °C (light gray, in the insert) incubation temperature the molluscicide was ineffective. *** = *p* < 0.001. Fisher’s exact test. In the case of a lack of nematode effects, no mortality was expected.

The biopesticide had dose-dependent effects on the mortality of apple snails at 18 and 25 °C, while at the 30 °C incubation temperature the molluscicide was always ineffective, independently of the dose applied (Figure 1). The treatments that affected snail mortality also diminished or annulled food consumption (Table 1). The 1.7 g/L dose was lethal for 30% and 14% of the snails when used at 18 and 25 °C, respectively. At 18 °C, the snails that survived until the end of the experiments never fed on the available salad, whereas at 25 °C the snails consumed only a limited amount of the food available. The 17 g/L treatment was lethal for all the snails at 18 °C and for 91% of specimens at 25 °C. During these experiments, the snails remained still in the tank, usually retracted into the shell, and never consumed the available food (Table 1). In line with the mortality count and feeding observations, a microscopic inspection of the tank water during daily water changes demonstrated that *P. hermaphrodita* specimens were alive and freely moving in tank waters at 18 and 25 °C. As previously documented [27], at 30 °C the microscopic observation of the tank waters revealed completely still worms (data not shown).

### 3.2. Histological Analysis of Nemaslug^®^-Treated Apple Snails

**Figure 2:**
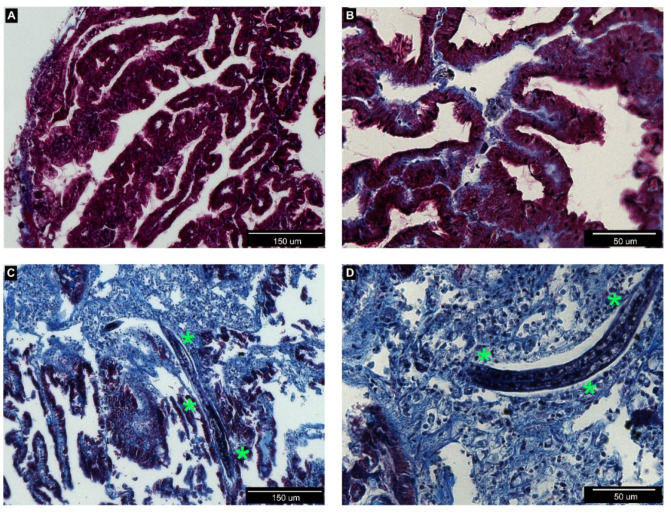
Histological sections of *P. canaliculata* anterior kidney collected from control (**A**,**B**) or treated (**C**,**D**) snails and stained by Masson’s trichrome staining. (**A**,**B**) In the anterior kidney, parenchyma is organized in ramified protrusions with a connective core and an external layer of epithelial cells. (**C**,**D**) A nematode is visible inside the renal parenchyma (green asterisks). No evident signs of encapsulation or hemocyte accumulation are visible.

**Figure 3:**
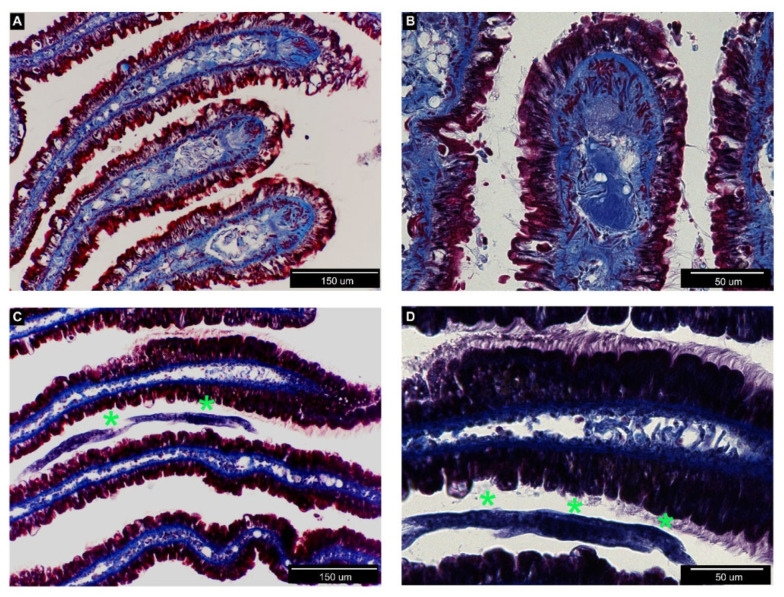
Histological sections of *P. canaliculata* gills (also known as ctenidia) collected from control (**A**,**B**) or treated (**C**,**D**) snails and stained with Masson’s trichrome staining. (**A**,**B**) Gills present parallel lamellae with a loose connective core (blue) and an external ciliated epithelium (red). (**C**,**D**) A nematode is visible between lamellae (green asterisks). (**D**) The gill epithelium remained intact and also at higher magnification the tissue organization and the cilia did not seem affected by the presence of the nematode.

**Figure 4:**
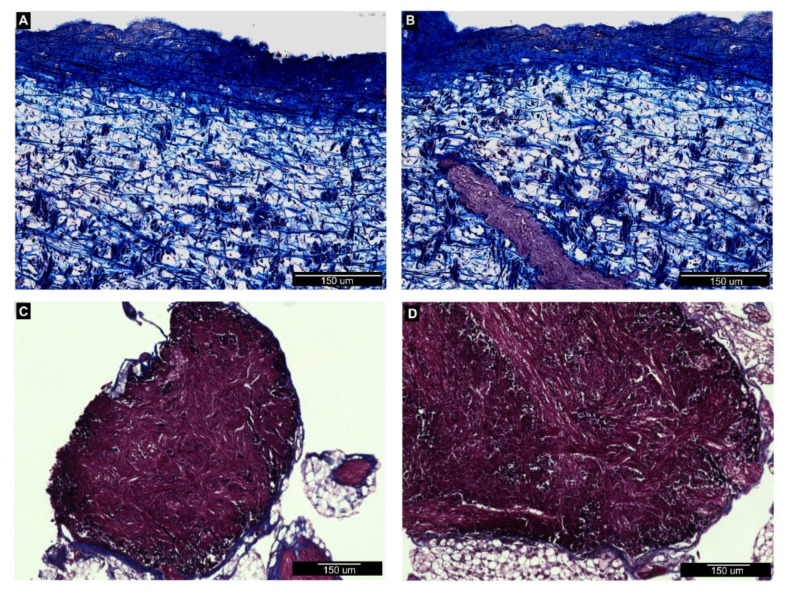
Histological sections of *P. canaliculata* (**A**,**B**) mantle and (**C**,**D**) pedal ganglia stained with Masson’s trichrome staining. (**A**). The mantle skirt tissue in control snails is mainly constituted by connective fibers, colored in blue, more densely packed in proximity of the external epithelium. (**B**) In treated snails, the mantle organization appeared well-conserved with no infiltration of nematodes. A portion of muscular tissue is visible in red. (**C**,**D**) A representative example of pedal ganglion showing highly dense neural tissue with neuron cell bodies and axons (red) clearly distinguishable from the peripheral connective tissue (blue). (**D**) Ganglia collected from treated snails display unaltered organization, and no *P. hermaphrodita* were observed either inside the neural tissue or close to the ganglion surface.

In order to document organ colonization by *P. hermaphrodita*, we analyzed histological sections of the anterior kidney (Figure 2), gills (Figure 3), mantle, and the cerebral and pedal ganglia (Figure 4) in animals incubated for one week with the recommended *P. hermaphrodita* concentration (i.e., 1.7 g/L) at 18, 25, and 30 °C. Masson’s trichrome staining unmasked the presence of nematodes in the anterior kidney and gills after incubations at 18 and 25 °C. The worms were able to penetrate the soft parenchyma of the anterior kidney (Figure 2C,D). Despite the presence of the nematode inside the organ, we observed neither a significant presence of hemocytes nor nodule formation. In the gills, *P. hermaphrodita* individuals adhered to the external surface of the epithelium, but failed to invade the organ (Figure 3C,D). Gill morphology remained similar to that of controls with no visible alterations in the epithelium adjacent to *P. hermaphrodita*. No worms were observed in the mantle or in the ganglia. Accordingly, the morphology of both organs was undistinguishable from that of control snails (Figure 4).

### 3.3. Pc-bpi Coding Sequence Identification and Analysis

In the attempt to identify plausible indicators of the activation of the snail immune response, we screened transcriptomic datasets generated from organs of adult *P. canaliculata* snails and, using this approach, we identified a sequence homologue to the *bpi* gene, that we called *Pc-bpi*.

The *Pc-bpi* sequence identified in the transcriptome and deposited in GenBank (accession number XM_025259146.1) encoded a 137 aa putative polypeptide, that shared 39% identity with *Cg*-BPI (accession number ACQ72935.1) identified in the oyster *Crassostrea* (*Magallana*) *gigas*, and 27% identity with the human BPI protein (accession number NP 001716.2).

**Figure 5:**
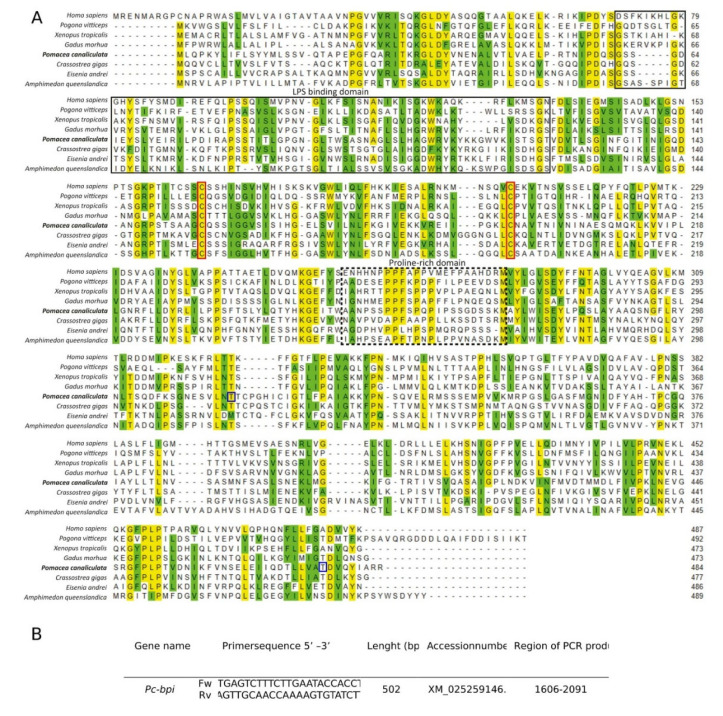
(**A**). Multiple sequence alignment of vertebrate and invertebrate BPI proteins using MUSCLE software. *Homo sapiens* (NP_001716.2), *Pogona vitticeps* (XP_020659320.1), *Xenopus tropicalis* (NP_001015694.1), *Gadus morhua* (AAM52336.1), *P. canaliculata* (XP_025114931.1), *Crassostrea gigas* (AAN84552.1), *Eisenia andrei* (AFI44048.1), and *Amphimedon queenslandica* (XP_019854892.1). Conserved amino acids are highlighted in yellow (identical amino acids with 70% consensus) and in green (similar amino acids with 70% consensus). The two conserved cysteine residues, putatively forming the disulfide bond, are boxed with red lines. The LPS-binding domain is boxed with a solid black line, and the proline-rich domain, connecting the C- and N-terminal domains, is boxed with a dashed black line. The blue box indicates the first and the last amino acids corresponding to the mRNA sequence targeted by the probe for in situ hybridization (**B**). Sequence of primers used for probe synthesis and essential information about the probe for in situ hybridization.

The analysis of functional domains in the *Pc*-BPI protein was performed on the HMMER website using the Pfam database. This revealed the presence of BPI conserved N- and C-terminal domains (PFAM reference numbers PF02886.17 and PF01273.25, respectively) with an independent e-value of 4.8e^−26^ and 3.1e^−45^ and a conditional e-value of 5.3e^−30^ and 3.4e^−49^, respectively. The Pc-BPI putative protein sequence also contained conserved cysteine residues that are found in all members of the LBP/BPI family [21] and are involved in the formation of a conserved disulfide bond, which is critical for protein structure, stability, and function [31,32]. The *Pc*-BPI putative peptide showed other important conserved residues, such as those in the region responsible for LPS binding (Figure 5).

### 3.4. Organ- and Temperature-Dependent Effects of Nemaslug^®^ on Pc-bpi mRNA Levels

**Figure 6:**
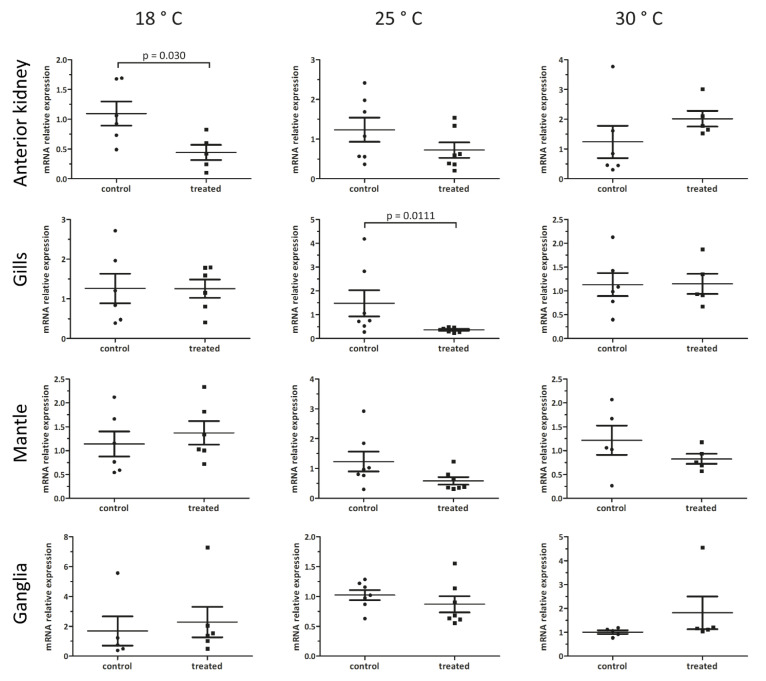
qPCR analysis of *Pc-bpi* expression performed on anterior kidney, gills, mantle, and ganglia of *P. canaliculata* at 18, 25, and 30 °C. The changes in mRNA expression between control and treated animals were calculated with the 2^-ΔΔCt^ method. *p*-value is reported only in the case of significant difference between control and treated samples. Each dot represents a single animal, bars represent mean +/- standard deviation.

In order to correlate the effects of the Nemaslug^®^ treatment on the survival and organ histology of snails with changes in gene expression indicative of immune activation, we monitored *Pc-bpi* expression in anterior kidney, gills, mantle, and the cerebral and pedal ganglia after 24 h of incubation with the 1.7 g/L of Nemaslug^®^ at 18, 25, and 30 °C. Our results show different temperature-dependent responses in the anterior kidney and gills. Indeed, when the snails were kept at 18 °C, *Pc-bpi* mRNA levels significantly decreased by 50% in the anterior kidney (*p* = 0.03), but not in the other organs (Figure 6). On the contrary, in animals treated at 25 °C, *Pc-bpi* mRNA amount decreased by 75% (*p* = 0.01) only in gills. No significant changes were observed in *Pc-bpi* mRNA levels when animals were exposed to the molluscicide at 30 °C (Figure 6).

### 3.5. FISH Analysis of Pc-bpi Expression in P. canaliculata Hemocytes

**Figure 7:**
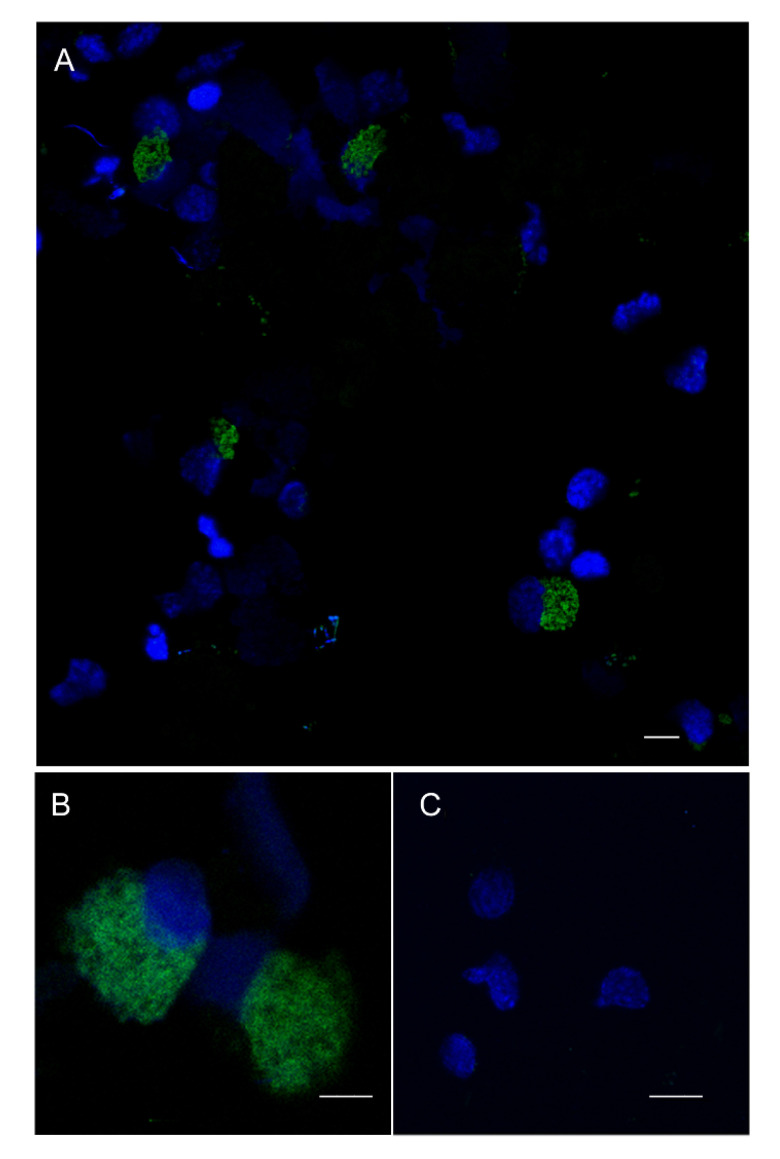
*Pc-bpi* constitutive expression in circulating hemocytes after cytocentrifugation and fluorescence in situ hybridization (FISH) experiments. (**A**) *Pc-bpi*-positive (green) hemocytes are evident. (**B**) Detail of positive hemocytes. (**C**) Negative control. Bars: (**A**) 5 µm,(**B**) 3 µm, (**C**) 4 µm.

The organs we have chosen for this study are characterized by a basal level of expression of *Pc-bpi* (Figure 6) and can be patrolled by resident or circulating hemocytes [6,9,10]. Therefore, we sought to address expression of *Pc-bpi* in circulating hemocytes using FISH. The automated cell detection protocol indicated that a minority of circulating hemocytes presented a clearly recognizable FISH signal and are therefore bona fide *Pc-bpi* expressing cells. Microscopy observations suggested that the positive hemocytes had a size resembling that of Group II hemocytes (Figure 7).

## 4. Discussion

The sustainable control of the spreading of the apple snail *P. canaliculata* is an important economical and health issue in numerous countries [3]. Here, we demonstrated that a *P. hermaphrodita*-based molluscicide reduces the survival of the apple snail. At the concentrations suggested by the manufacturer, commercially available *P. hermaphrodita*-based molluscicide stops the snails feeding rapidly and efficiently, a fundamental goal for crop producers. Further studies on the long-term effects on snail feeding will help to clarify whether the impact of the molluscicide on snail feeding is reversible. *P. hermaphrodita* at the concentration of 30 (i.e., the recommended dose) and 150 worms/cm^2^ has been reported to be ineffective towards the freshwater snails *Lymnaea stagnalis, Planorbarius corneus*, and *Bithynia tentaculata*, both in terms of survival and weight gain in a 66-day long experiment [19]. In our 1-week experiment, we also observed a lack of effect at the lowest tested concentration, but increasing the concentration of the biopesticide resulted in a dose-dependent killing of the apple snails. Additionally, among terrestrial snails and slugs, significant differences in the susceptibility to the nematode have been reported [17,18], outlining the necessity of further studies to clarify the *P. hermaphrodita* mechanism of action in aquatic and terrestrial gastropods. In the land slug *Deroceras reticulatum*, *P. hermaphrodita* juvenile larvae invade the dorsal integumental pouch, immediately posterior to the mantle of the slug. Here they develop into sexually mature adults, reproduce as self-fertilizing hermaphrodites, leading to the slug’s demise [33,34] through yet ill-defined mechanisms. In one research report, it was indicated that the lethality observed in the land slug *D. reticulatum* after exposure to the worms could be caused by the Gram-negative microbe *Moraxella osloensis*, associated with the nematode in commercially reared stocks [34]. Further studies however reported that, in saprobic culture, the bacteria species associated with the nematode change in different co-culture experiments, without affecting virulence [35]. Beside the direct pathogenicity of the worms and the associated bacteria, other components could explain the different effects of *P. hermaphrodita*, including the capability of some snails to utilize their shell as a defense tool [36] or the ability of the worm to interfere with the behavior of the mollusk [37]. In these respects, our treatments performed at 30 °C were ineffective at all the tested concentrations of the molluscicide. In accordance with previous observations [27], the nematode viability is compromised at higher temperature, suggesting that dauer forms or dead *P. hermaphrodita* are unable to damage *P. canaliculata*, independently of the presence of pathogenic bacteria. This observation points towards a direct effect of the nematode [35].

As the details of *P. hermaphrodita* pathogenicity have not been elucidated and may differ between aquatic and land species of gastropods, we performed a histological analysis of some potential target organs. Toward this end, we prioritized the analysis of organs that are notoriously linked with the immune functions, like the anterior kidney [8] and the gills [9]. Our histological observations did not evidence nematodes infiltrated within the mantle skirt or in the depth of neural ganglia. Moreover, in organs where the nematode was detected, such as the anterior kidney and the gill epithelium, the presence of the worm was not associated with observable tissue alterations, and we observed neither hemocyte accumulation nor nodulation, on the contrary of what has been reported for *Pomacea* lungs after *A. cantonensis* infection [14].

In order to evaluate whether *P. canaliculata* could raise a detectable immune response when exposed to recommended doses of a *P. hermaphrodita*-based molluscicide, and in the absence of well-established readouts of immune system activation like those observed in other invertebrate models [38], we looked for a *bona fide* antimicrobial peptide candidate to be investigated by qPCR and serving as a potential readout of immune system activation. By searching organ-specific RNAseq databases and genomes [1,2], we identified Pc-BPI, a likely member of the LPB/BPI family in *P. canaliculata*. Functional studies on BPI proteins in the oyster *C. gigas* [39,40] showed that LBI/BPI family members share structural similarities with the mammalian LBP/BPIs, including conserved bactericidal and membrane-permeabilizing effects against Gram-negative bacteria. The planorbid *Biomphalaria glabrata*, a well-studied freshwater gastropod due to its role as intermediate host for parasite flatworms, and relatively close to *P. canaliculata* [1], contains five LBP/BPI homolog sequences [24,41]. This suggests a serial duplication of the LBP/BPI locus that could have led to a diversification and functional specialization of the different proteins, although they all have conserved the N- and C-terminal domains characteristic of the mammalian LBP/BPIs, as well as the amino acidic residues critical for their structure and function [21]. Sequence and domain analyses suggest that *Pc*-BPI may also be endowed with antimicrobial activity, and we analyzed its expression by qPCR in control snails as well as after exposure to Nemaslug^®^. At the recommended concentration of 1.7 g/L, the molluscicide promotes a significant decrease in the expression of *Pc-bpi* in the anterior kidney, at 18 °C, and in the gills, at 25 °C. In agreement with the absence of morphological alterations in mantle and ganglia, these organs did not present significant changes in *Pc-bpi* expression. A selective reduction in mRNA levels of an immune-related molecule after injection of bacterial LPS has already been documented in the anterior kidney, gills, and mantle of *P. canaliculata*, whereas other organs, e.g., pedal ganglia, remained unaffected [8]. Our data collected after *P. hermaphrodita* infection are in line with the organ-specific immune response in *P. canaliculata*, highlighting, in this case, the involvement of the anterior kidney and the gills, organs that have been reported as main players of the apple snail immune response [8,9] and that express genes involved in xenobiotic metabolism and cell growth [1]. Accordingly, a close connection between the anterior kidney and the gills has been described, both in terms of anatomical proximity and blood (i.e., hemolymph) flux [10]. In this context, we observed that circulating hemocytes can display a basal expression of *Pc-bpi*. The absence of specific hemocyte markers does not allow to conclude if the levels of expression we registered by qPCR in control animals can be attributed to resident or circulating hemocytes patrolling those organs [6,9,10]. However, in the absence of univocal hemocyte markers [16], it cannot be ruled out that the changes we observed in the anterior kidney and the gills might be also due to changes occurring in the resident hemocyte population [6,9,10].

Our real-time PCR experiments also evidenced a correlation between the organ-specific response and experimental temperature. Since the 18–25 °C temperature range is optimal for the apple snail survival and reproduction [26], it is possible that the temperature-related effects reflect a different capability of the nematode to engage the snails’ immune defense at 18 and 25 °C. In these respects, our histological observations detected the presence of the nematodes in the anterior kidney and gills at both 18 and 25 °C. However, at this stage, a differential, temperature-dependent immune response of the snail cannot be ruled out. In bivalves, the correlation between water temperature and mollusk susceptibility to stress and infections has been well documented [42,43,44,45]. In the freshwater snail *L. stagnalis*, it has been observed that changes in environmental conditions may alter the activity of several immune components. Moreover, cellular and humoral components can respond differently to temperature changes as well as the duration of the treatment [46,47,48]. Environmental temperature is one of most critical parameters influencing *P. canaliculata* reproduction and life span [49], and temperature-dependent changes in the expression of molecules of the heat shock protein (HSP) family has been demonstrated for gills and kidney [50].

Beside reporting the potential utilization of a commercial molluscicide for the sustainable control of the diffusion of this invasive snail, our data indicate that the commercial formulation of *P. hermaphrodita* is a useful experimental tool for investigating *P. canaliculata* immunity. The vast majority of experiments mimicking an immune challenge in *P. canaliculata* are based on the injection of potential pathogens [10] or bacterial extracts [8,12]. While pathogen injection provides the experimenter with a tight control over the dose of the stimulants, thus reducing inter-individual variability [51], this approach is not the natural way of access for a pathogen in aquatic environments and it artificially overcomes the natural immune barriers of the snail. Here, we provide evidence that, in contact with a naturally occurring pathogen, the response of the involved organs changes at different temperatures. Whether the temperature-correlated effects rely more on worm fitness, or on temperature-dependence of immune reaction in the apple snail, or a combination of both, remains to be established, but our approach could better reproduce the natural dynamic between pathogens and the host in natural environments. We also reason that this dynamic is likely to underline the interaction between the apple snail and the human parasite *A. cantonensis*. In these respects, our findings contribute towards a pioneering elucidation of *P. canaliculata* immune components [8,9,10] and warrant further investigations to characterize the complex immune features and organ-specific functions that mediate *P. canaliculata* response to pathogenic assaults.

## Figures and Tables

**Table 1:** Effects of different concentrations of Nemaslug^®^ on *Pomacea canaliculata* daily food intake at different temperatures for 1 week.

Concentration (g/L)	Temperature (°C)	Food Intake
0.17	18	+
	25	+
	30	+
1.7	18	-
	25	+/-
	30	+
17	18	-
	25	-
	30	+

+ = all the salad (approximately 10 g) was consumed within 24 h. +/- = salad was only partially consumed. - = salad was left untouched within 24 h.
